# Tumor-selective peptide-carrier delivery of Paclitaxel increases *in vivo* activity of the drug

**DOI:** 10.1038/srep17736

**Published:** 2015-12-02

**Authors:** Jlenia Brunetti, Serena Pillozzi, Chiara Falciani, Lorenzo Depau, Eleonora Tenori, Silvia Scali, Luisa Lozzi, Alessandro Pini, Annarosa Arcangeli, Stefano Menichetti, Luisa Bracci

**Affiliations:** 1 University of Siena, Department of Medical Biotechnologies, Siena, 53100, Italy; 2 University of Florence, Department of Experimental and Clinical Medicine, Florence, 50139, Italy; 3 SetLance srl, Toscana Life Sciences, Siena, 53100, Italy; 4 University of Florence, Department of Chemistry, Florence, 50019, Italy

## Abstract

Taxanes are highly effective chemotherapeutic drugs against proliferating cancer and an established option in the standard treatment of ovarian and breast cancer. However, treatment with paclitaxel is associated with severe side effects, including sensory axonal neuropathy, and its poor solubility in water complicates its formulation. In this paper we report the *in vitro* and *in vivo* activity of a new form of paclitaxel, modified for conjugation with a tumor-selective tetrabranched peptide carrier (NT4). NT4 selectively targets tumor cells by binding to membrane sulfated glycosaminoglycans (GAG) and to endocytic receptors, like LRP1 and LRP6, which are established tumor markers. Biological activity of NT4-paclitaxel was tested *in vitro* on MDA-MB 231 and SKOV-3 cell lines, representing breast and ovarian cancer, respectively, and *in vivo* in an orthotopic mouse model of human breast cancer. Using *in vivo* bioluminescence imaging, we found that conjugation of paclitaxel with the NT4 peptide led to increased therapeutic activity of the drug *in vivo*. NT4-paclitaxel induced tumor regression, whereas treatment with unconjugated paclitaxel only produced a reduction in tumor growth. Moreover, unlike paclitaxel, NT4-paclitaxel is very hydrophilic, which may improve its pharmacokinetic profile and allow the use of less toxic dilution buffers, further decreasing its general chemotherapic toxicity.

Taxanes, which include paclitaxel, docetaxel and other analogues currently under clinical trial, are a class of potent chemotherapeutic drugs that act by binding microtubules and preventing their disassembly, thus blocking mitosis and eventually inducing apoptosis. Taxanes are highly effective against proliferating cancer and are an established option in the standard treatment of ovarian and breast cancer, despite two major drawbacks, which they share with most chemotherapy drugs: drug resistance^1^ and toxicity^2^. In the case of taxanes, in addition to the toxicity of the drug itself, further side effects have been associated with dilution solvents, needed because of high taxane hydrophobicity^3^,^4^.

Sensory axonal neuropathy is among the worst side effects associated with taxanes and has an incidence of up to 30%^5^. Neurological symptoms can be so severe as to necessitate discontinuation or reduction of treatment. Although symptoms usually resolve after the end of treatment, they can persist for months or years, spoiling quality of life^6^. Severe blood toxicity has also been associated with taxane treatment^5^. The toxic effects of taxanes are experienced by a large number of cancer patients throughout the world and are a major physical, psychological and financial burden for patients and society^7^.

Breast cancer is the most common cancer in women worldwide and the second most frequent type of cancer in the whole population, accounting for 15% of all cases, with more than 1,676,000 new cases diagnosed in Europe in 2012^8^ and an 85% five-year survival worldwide^9^.

Ovarian cancer is the fifth most diagnosed tumor in Europe, accounting for 2% of all cancer cases and 3% of all cancer deaths^10^. Women diagnosed with ovarian cancer in 2005–2009 in USA and Europe have a five-year survival of about 40%. Improved treatment of breast and gynecological tumors is a concrete medical need.

Here we report the *in vitro* and *in vivo* activity of a new form of paclitaxel, modified for conjugation with a tumor-selective tetrabranched peptide carrier.

We know from foregoing studies that unlike the parent linear peptides, tetrabranched peptides synthesized on a three-lysine core are extremely resistant to biological degradation by peptidases, and may maintain or even increase their biological activity through multimeric binding^11^. They are therefore much more interesting than linear peptides for development as drugs^12^,^13^.

In previous papers, we synthesized tetrabranched NT peptides (NT4) conjugated with different functional units for selective imaging and killing of cancer cells^14^,^15^,^16^. We demonstrated that NT4 peptides efficiently discriminate between tumor and healthy tissue in human surgical samples of colon adenocarcinoma, pancreas adenocarcinoma and bladder cancer from a large series of patients, with good statistical significance^15^,^16^. We also proved that NT4 can efficiently and selectively steer functional units^17^ or liposomes^18^ for cell imaging or killing of different human cancer cells. Using NT4 conjugated with methotrexate or 5FdU, we obtained a significant reduction in tumor growth in mice^14^,^15^.

We also demonstrated that the high cancer selectivity of NT4 peptides is due to their multimericity, which allows binding to membrane sulfated glycosaminoglycans (GAG) as well as to different membrane endocytic receptors of the low density lipoprotein receptor (LDLR) family, like LRP1 and LRP6, which are already known as potentially druggable tumor markers involved in many aspects of cancer biology. Systematic modification of amino acid sequence in NT4 peptides led to identification of a multimeric, positively charged motif that mediates interactions with heparan sulfated GAGs and LRP receptors^19^. Conjugation of paclitaxel with the NT4 peptide led to increased therapeutic activity of the drug in an orthotopic model of breast cancer in mice.

## Results

### Synthesis of NT4-paclitaxel

**Figure 1:**
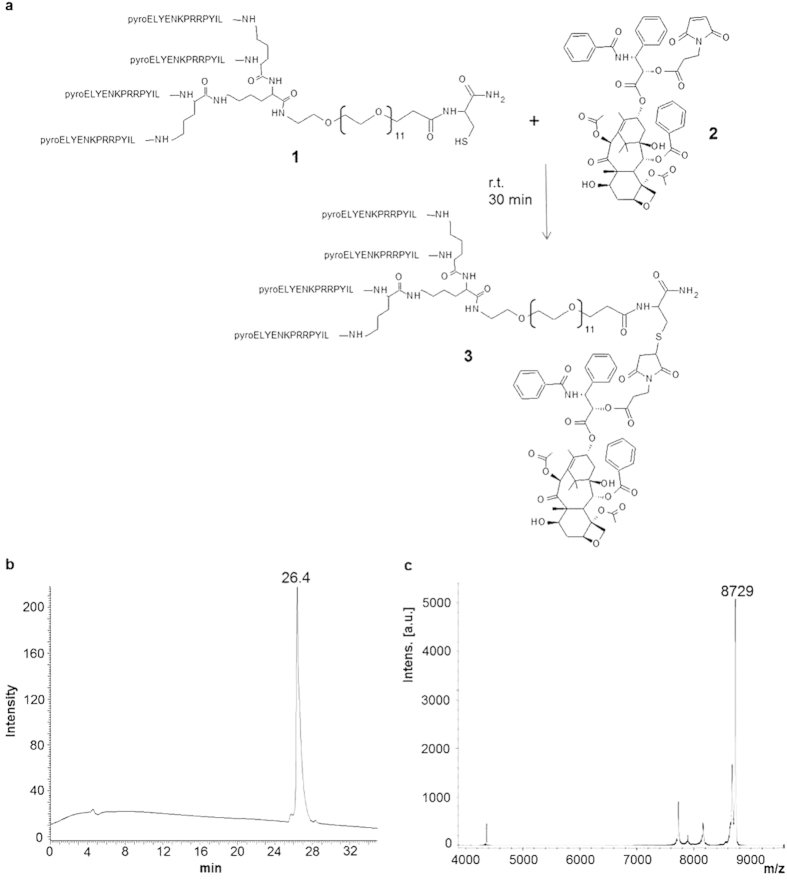
Synthesis (**a**) and purification of the tetrabranched peptide conjugated with paclitaxel (NT4-PTX). HPLC **(b)** and MS **(c)** profiles of NT4-PTX. UV chromatogram at 220 nm of the purified NT4-PTX peptide, obtained by using reverse phase HPLC on a Jupiter C18 analytical column **(b)**. NT4-PTX MALDI-TOF spectrum after purification **(c)**.

The NT4 tetrabranched peptide was synthesized by solid phase and conjugated with paclitaxel to obtain a tumor-targeting moiety linked to a well established chemotherapeutic. Paclitaxel was adapted to the orthogonal conjugation with a maleimide-carboxy bifunctional linker (Fig. 1). Identity and purity of the final product, NT4-PTX, was confirmed by analytical reverse phase chromatography on a Jupiter C18 column (Fig. 1b) and mass spectrometry (MS) (Fig. 1c).

The product was more than 90% pure, as shown by HPLC (26.4 retention time) and MS, revealing a peak at the expected molecular mass (8,729 Da).

### NT4 binding to ovarian and breast cancer cells

**Figure 2:**
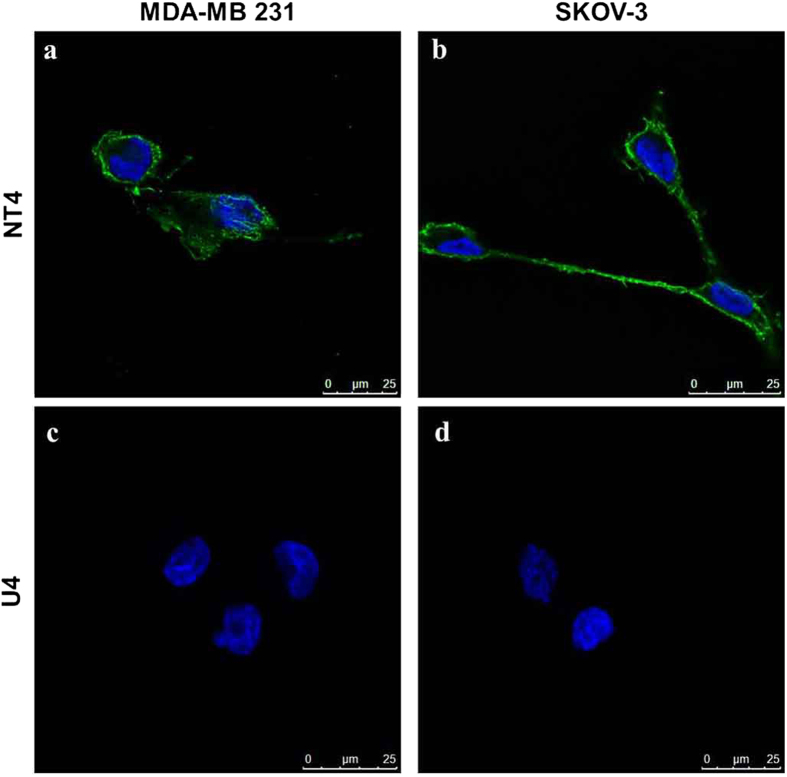
Binding of NT4 peptide (**a,b**) and the unrelated tetrabranched peptide U4 (**c,d**) to human breast and ovarian cancer cell lines. NT4 conjugated with biotin and then incubated with streptavidin-FITC (green) bound to MDA-MB 231 **(a)** and SKOV-3 **(b)** cells. The unrelated tetrabranched peptide U4-biotin (**c**,**d**) did not show any binding. Nuclei stained with DAPI (blue).

Binding of fluorophore-conjugated tetrabranched NT4 was tested in two human cell lines, namely SKOV-3 ovarian adenocarcinoma and MDA-MB 231 breast adenocarcinoma. NT4 peptide, conjugated with biotin and traced with streptavidin-FITC, bound MDA-MB 231 and SKOV-3 cells (Fig. 2a,b). A control tetrabranched peptide (U4) with an unrelated amino acid sequence, was used to exclude unspecific fluorescence (Fig. 2c,d). These results are in line with previous findings obtained with human colon, pancreas and bladder tumor cell lines under the same experimental conditions. NT4 selectivity to tumor cells was previously described^15^,^16^ in tumor versus healthy tissue from human surgical specimens.

### Paclitaxel release

**Figure 3:**
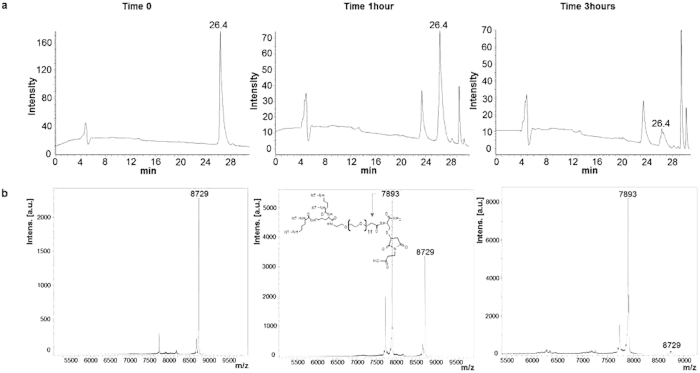
Paclitaxel release. HPLC (**a**) and MS (**b**) profiles of NT4-PTX incubated with 3% serum at different times: 5 min, 1 hour and 3 hours. The structure reported in the panel of MS analysis at time 1 hour, corresponds to the fragment where PTX is released by hydrolysis of the ester bond.

Release of the chemotherapeutic from the adduct was assessed with the tetrabranched NT4 peptide (NT4-PTX; Fig. 3) and with an unreleated tetrabranched peptide (U4-PTX; supplementary material Fig. S1) conjugated with paclitaxel. NT4-PTX was incubated in 3% serum for different time intervals, 5 min (time 0), 1 h and 3 h, as described previously^11^. Presence of the uncleaved peptide NT4-PTX (molecular weight 8,729 Da) was checked by HPLC and MS. The paclitaxel moiety was released after one hour of incubation, as reported in Fig. 3. The NT4-maleimide peak (molecular weight 7,893 Da) appeared after one hour with a shorter retention time than the intact peptide. The drug was almost completely released in 3 hours.

### Cytotoxicity of NT4-PTX

**Figure 4:**
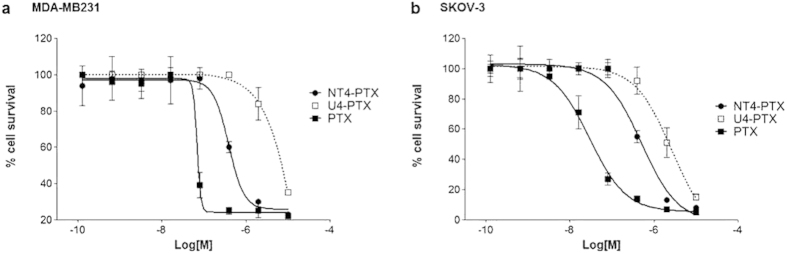
Cytotoxicity of NT4 peptide conjugated with paclitaxel in MDA-MB 231 (**a**) and SKOV-3 (**b**) cell lines. Cytotoxicity of NT4-PTX was compared with that of the corresponding free drugs (PTX) and an unrelated tetrabranched peptide (U4-PTX), conjugated identically with the same drug. EC50: NT4-PTX, 3.8 × 10^−7^M and 4.0 × 10^−7^M; free PTX, 6.2 × 10^−8^M and 2.9 × 10^−8^M; U4-PTX, 2.1 × 10^−6^M and 2.0 × 10^−6^M, in MDA-MB 231 and SKOV-3, respectively. p < 0.05 for two-sided testing.

The cytotoxicity of NT4-PTX was tested in MDA-MB 231 and SKOV-3 human cancer cell lines (Fig. 4). Cellular toxicity of NT4-PTX was compared to that of the corresponding free drug (PTX) and to that of the unrelated tetrabranched peptide (U4-PTX), which had been synthesized and conjugated with paclitaxel by procedures identical to those used for synthesis and conjugation of NT4-PTX, though with a different, non-tumor-selective amino acid sequence. In order to compensate the effect of free PTX, which is released from the peptides by hydrolysis of the ester bond, cells were incubated for 1 hour and then washed and incubated without any drug for 6 days.

The EC50 of NT4-PTX was 3.8 × 10^−7^M and 4.0 × 10^−7^M and that of free PTX was 6.2 × 10^−8^M and 2.9 × 10^−8^M in MDA-MB 231 and SKOV-3, respectively. The level of significance (Student’s t test) of the cytotoxicity experiments was analyzed by comparing the data sets of NT4-conjugated peptides with the free drugs and with conjugated unrelated peptides (p < 0.05 in all cases).

Results obtained with the control U4-PTX unrelated peptide conjugate showed that conjugation with a branched peptide that does not bind to cancer cells^15^ produces a reduction in paclitaxel activity, thus confirming that the cytotoxicity of NT4-PTX is due to its peptide-mediated transport into cancer cells. No cytotoxic effect was measured using unconjugated NT4 at the same concentrations (not shown).

The tumor-specific internalization process of NT4-PTX provides the advantage of selectivity to tumor versus healthy cells, which can be better appreciated in *in-vivo* assays were many different type of cells are represented.

### Tumor growth in an orthotopic mouse model

NT4-PTX efficacy was tested *in vivo* in an orthotopic mouse model. MDA-MB 231-*luc2* cells were injected orthotopically in the mammary fat pads of female athymic nude mice. Once mice attained palpable tumors, they were randomly divided into three groups and treated with NT4-PTX (2.86 μmol/Kg), free PTX (2.86 μmol/Kg) and saline every 4 days by tail vein injection. Tumor growth was monitored by measuring volumes every 4 days with a caliber rule and by imaging luciferase reporter expression in tumor cells with a Photon Imager system at 8-day intervals.

**Figure 5:**
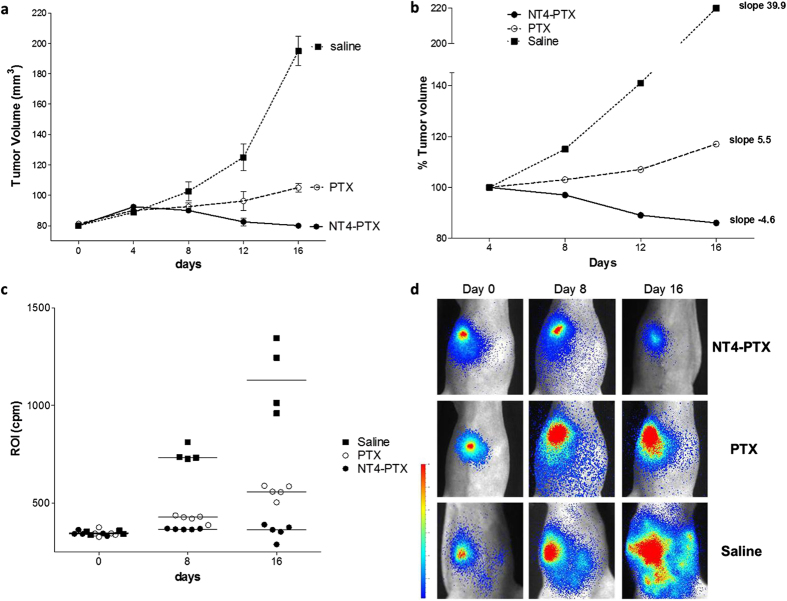
Effect of NT4-PTX and PTX on orthotopic MDA-MB 231-*luc2* tumor growth. **(a)** Female athymic mice bearing 80 mm^3^ MDA-MB 231-*luc2* tumors were treated i.v. with NT4-PTX (group 1, n = 5 mice), free PTX (group 2, n = 5 mice ) and saline (group 3, n = 4 mice) every four days. Tumor volume was calculated using the formula *(a*^*2*^ × *b*)/2, where *a* and *b* are perpendicular diameters, *a* is the width of the tumor (small diameter), and *b* the length (large diameter), both expressed in millimeters. **(b)** Average tumor volumes were reported as a percentage of the volume at day 4, when tumor volumes were almost identical in the three groups. **(c**,**d)** *In vivo* tumor imaging. The animals were injected i.p. with D-luciferin and then imaged with a Photon Imager system. Mice that received different treatments were imaged at various time points (0, 8 and 16 days after the first dose). **(c)** The line represents the mean ROI value for each group. **(d)** Images of the most representative animals are shown for each treatment group.

At day 4 after the first injection, the effect of the drugs was not yet detectable since average tumor volumes, as measured with the caliber were similar in the three groups of mice. In the following days, tumor growth, as measured by caliber and imaging, was significantly lower in mice treated with NT4-PTX than in control mice and mice treated with unconjugated PTX. At day 16, the average tumor volume of mice treated with NT4-PTX, as measured by caliber and imaging, had decreased by about 37% with respect to animals treated with free PTX and by 70% with respect to control animals (saline) (Fig. 5a). In order to better visualize tumor growth during treatment, average tumor volumes were reported as a percentage of the volume on day 4, when tumor volumes were almost identical in the three groups. The slopes of the curves obtained plotting average tumor volumes on days 4, 8, 12 and 16 clearly showed tumor regression, indicated by a negative slope, in the group of animals treated with NT4-PTX, whereas treatment with unconjugated PTX produced a clear reduction in tumor growth with respect to controls, but not tumor regression (Fig. 5b).

Figure 5c shows tumor bioluminescence signals in treated animals. Images from the most representative animal of the group is shown for each treatment group (Fig. 5d).

**Figure 6:**
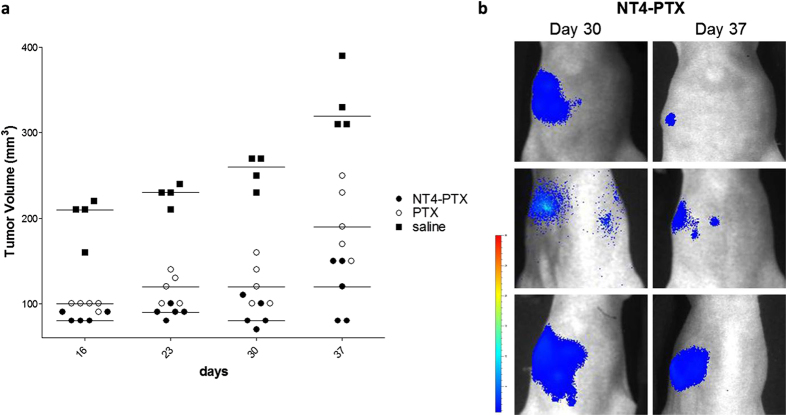
The effect of NT4-PTX and PTX on orthotopic MDA-MB 231-*luc2* tumor growth after the last treatment. **(a)** Tumor volume of mice treated with PTX and NT4-PTX, monitored for three weeks after the last drug injection. The line represents the mean ROI value for each group. **(b)** Images at day 30 and 37 of the three animals in the group treated with NT4-PTX that showed almost complete eradication of the disease.

Mice treated with PTX and NT4-PTX were monitored for at least 2 weeks after the last drug injection. Among animals treated with NT4-PTX, regression was observed in each treated animal and 3/5 treated animals showed almost complete eradication of the disease (Fig. 6) with no recurrence during 3 weeks without treatment.

Toxicity of NT4-PTX treatment was evaluated by monitoring animals daily. None of the treated animals showed any remarkable change in behavior or significant loss of body weight.

## Discussion

Paclitaxel is considered one of the most promising cancer chemotherapy drugs and has been tested for many different human malignancies. Taxanes, like paclitaxel, stabilize microtubules preventing their depolymerization during cell mitosis, which eventually leads to cell death. The mechanism of action is therefore targeted essentially at dividing cells. However, treatment with paclitaxel is associated with severe side effects. Being hydrophobic, taxanes can be transported passively into practically any cell. Though neurons do not divide, they are also susceptible to paclitaxel and this complicates its use as a therapeutic agent. Moreover, clinical development has been impeded by the very high hydrophobicity of paclitaxel and related difficulties with its formulation. Many side effects associated with treatment with paclitaxel have also been ascribed to dilution solvents used in the formulation^4^. Various strategies have been developed to obtain new analogues or new formulations of taxanes with increased cancer cell selectivity, reduced toxicity and improved pharmacokinetic profile^3^,^4^,^20^.

Here we report a new paclitaxel-carrier conjugate, NT4-PTX, in which the drug is conjugated with the highly hydrophilic tetrabranched NT4 peptide, already reported in previous studies as a tumor-selective peptide carrier^14^,^15^,^16^. NT4-PTX activity was tested *in vitro* on MDA-MB 231 and SKOV-3 cell lines, representing breast and ovarian cancer respectively, and *in vivo* in an orthotopic mouse model of human breast cancer. When conjugated with NT4 peptide, paclitaxel was active against both the breast and ovarian cancer cell lines and had less activity than the unconjugated drug. The EC50 of NT4-PTX was essentially in line with that measured with different previously tested NT4-drug conjugates^14^,^15^,^16^,^17^. PTX exerts its toxicity after efficiently, but non-selectively, crossing the cell membrane, whereas NT4-PTX is specifically internalized in tumor cells. This difference in the internalization process may be responsible for the lower *in vitro* efficiency of NT4-PTX, but it also confers to NT4-PTX the advantage of selectivity to tumor versus healthy cells, which cannot be appreciated in *in-vitro* assays were only a single cell type is present.

Since NT4 selectivity for cancer cells cannot be exploited in *in vitro* cytotoxicity tests on cancer cells alone, we compared the cytotoxicity of NT4-PTX with the unconjugated drug in an *in vivo* animal model.

To compare antitumor activity of NT4 peptide conjugated with PTX with that of the unconjugated drug, we used an orthotopic nude mouse model with the MDA-MB 231-*luc2* human breast cancer cell line. Breast cancer cell engraftment into the mammary fat pad of mice reproduces the location of the disease with a proper stromal compartment, therefore mimicking cancer in humans.

Two groups of mice were injected intravenously with equimolar concentrations of NT4-PTX or unconjugated PTX and a third group was injected with saline as control. At day 4 after injection, average tumor volumes in the three groups were still almost identical, indicating that drug activity was not yet detectable. Tumor growth in the three groups was subsequently different. Mice treated with PTX showed a clear reduction in average tumor growth with respect to controls, though with no tumor regression, as indicated by a positive slope of the curve obtained by plotting average tumor volume variation. Interestingly, growth of tumors in the group of mice treated with NT4-PTX showed a negative slope during treatment, indicating tumor regression not obtained with unconjugated paclitaxel. Despite lower *in vitro* activity of NT4-PTX with respect to the free drug, the animal model experiments indicated higher *in vivo* activity of NT4-PTX. The increase of *in vivo* activity of PTX induced by conjugation with NT4 peptide may be related to its higher cancer selectivity. The active *in vivo* concentration of the free drug at the tumor site is in fact decreased by nonselective uptake by non-tumor cells. Conjugation with NT4 may mediate binding and internalization only to cells that over-express NT4 membrane receptors. Moreover, unlike PTX, NT4-PTX is very hydrophilic. This may improve its pharmacokinetic profile and enable less toxic dilution buffers to be used, thus further decreasing the general toxicity of chemotherapy. Our results suggest that NT4-mediated targeted paclitaxel therapy may not only be less toxic, but also more effective.

## Methods

### Synthesis - General

All the reactions were monitored by TLC on commercially available precoated plates (silica gel 60 F 254) and the products were visualized with acidic vanillin solution.

Silica gel 60, 230–400 mesh, was used for column chromatography, unless otherwise stated. EtP stands for light petroleum, bp 40–60 °C, and EtOAc for ethylacetate. ^1^H and ^13^C NMR spectra were recorded at 400, 200, 100 and 50 MHz, respectively. Melting points were measured on a microscopic apparatus and are uncorrected. FTIR spectra were recorded in KBr pellets or CDCl3 solutions. Mass spectra were measured with a Shimadzu QP5050, by FAB (m-nitrobenzyl alcohol as matrix) or by ESI using JEOL MStation JMS700.

Tetrahydrofuran was distilled from sodium in the presence of the blue colour of benzophenone ketyl; toluene was distilled from sodium, CH_2_Cl_2_ from CaH_2_ and MeOH from Mg.

Solid-phase synthesis was carried out on a MultiSynTech Syro automated multiple peptide synthesizer (Witten, Germany), employing Fmoc chemistry with 2-(1H-benzotriazole-1-yl)-1,1,3,3-tetramethyluronium hexafluorophosphate/N,N-diisopropylethylamine (HBTU) activation and NovaSyn TGR (Novabiochem). Side chain protecting groups were trityl for His and Asn, 2,2,4,6,7-pentamethyldihydrobenzofuran-5-sulfonyl (Pbf) for Arg, tert-butyl ether (tBu) for Ser and Tyr, tert-butyl ester (OtBu) for Asp and Glu, and tert-butyloxycarbonyl (Boc) for Lys. Peptides were cleaved from the resins and deprotected by treatment with trifluoroacetic acid containing water and triisopropylsilane (95:2.5:2.5) for 1.5 hours at room temperature. After precipitation with diethyl ether, branched peptides were purified by RP-HPLC. Final peptide purity was confirmed to be over 99% by HPLC on a C18 Jupiter column (Phenomenex, 300 Å, 5 μm, 250 × 4.6 mm) using 0.1% TFA/water as eluent A and methanol as eluent B with a linear gradient from 80% A to 5% A in 30 min. All peptides were characterized by UltraflexIII MALDI TOF/TOF mass spectrometry (Bruker Daltonics, Bremen, Germany). Commercial reagents, catalysts and ligands were used without further purification from freshly opened containers, unless otherwise stated.

### Synthesis of 2′-maleimide-paclitaxel

DMAP (0.0007 g, 0.006 mmol) was added to a solution of Paclitaxel (0.050 g, 0.06 mmol) in 8 mL dry DCM under a N_2_ atmosphere. The colourless solution was cooled to 0 °C, then 3-maleimidopropionic acid (0.01 g, 0.06 mmol) was added, followed by DIC (9 μL, 0.06 mmol). The reaction mixture was stirred for 18 hours at room temperature, producing a reddish solution, then poured onto 20 mL DCM and washed with a saturated solution of NH_4_Cl (3 × 40 mL), then with a saturated solution of NaHCO_3_ (2 × 40 mL) followed by a saturated solution of NH_4_Cl (5 × 40 mL). The organic layer was dried over Na_2_SO_4_, filtered and evaporated to obtain a reddish oil (48 mg). The crude product was purified by flash chromatography using EtP:AcoEt = 2:3 as eluent to obtain the final pure product as white powder with 46% yield. ^1^H NMR, 400 MHz, CDCl_3_^21^. ^13^C-NMR, 100 MHz, CDCl_3_, δ: 9.59; 14.77; 20.80; 22.14; 22.67; 26.77; 32.47; 33.29; 35.48; 43.13; 45.54; 52.36; 58.49; 71.74; 72.09; 74.47; 75.12; 75.60; 79.14; 81.02; 84.45 (24C, Caliph.) 126.52; 127.43; 128.27; 128.50; 128.72; 129.01; 129.22; 130.26; 131.87; 132.67; 133.64; 134.03; 136.87; 142.86 (22C, Carom+ 2C = C); 167.04; 167.24; 167.68; 169.36; 169.88; 171.24 (7C, C = O); 203.83 (1C, C = Oketone). IR (CDCl_3_), cm-1: 3607.75, 3517.24 (m, OH stretch. free and bonded); 3387.93 (m, NH stretch. bonded); 3060.96 (w, CHarom stretch.); 2948.27, 2896.55 (w, CHaliph. stretch.); 1737.63 (s, C = Oester stretch.); 1711.82 (s, C = Oketone stretch.); 1664.51 (s, C = Oamide stretch.). ESI -MS: m/z  =  1027.33 [M+Na]+; 1039.67 [M+Cl].

### Synthesis of NT4-PTX, U4-PTX and NT4-biotin

NT4-PTX and U4-PTX were synthesized on Novasyn TGR resin using Fmoc-Cys(Trt)-OH as first coupling step, and Fmoc-PEG_12_-OH (Iris Biotech, Germany) as second. Then two coupling steps with Fmoc-Lys(Fmoc)-OH were used to build the core. For NT4-PTX, Pyro-Glu-OPentachlorophenylester was used for the N-terminal acid of the neurotensin sequence. Peptides were cleaved from the resin and deprotected with 95% TFA. After purification by HPLC, the branched-thiol intermediate was dissolved in DMF (500 μM), mixed with a solution of 2′-maleimide-paclitaxel in DMF (5 mM) (10 Equivalents) and 0.1% v/v DIPEA. The reaction was stirred for 30 minutes at r.t., then purified by HPLC.

NT4-biotin was synthesized using Fmoc-Lys-(Biotin)-OH (Iris Biochem GmbH, Marktredwitz, Germany) as first group coupled to the solid phase and Fmoc-PEG_12_-OH (Iris Biochem GmbH) as second.

### Cell cultures

MDA-MB 231 human breast adenocarcinoma and SKOV-3 human ovary adenocarcinoma were grown in Leibovitz’s L-15 Medium and McCoy’s 5a Medium, respectively, supplemented with 10% fetal bovine serum, 200 μg/ml glutamine, 100 μg/ml streptomycin and 60 μg/ml penicillin. Only SKOV-3 were maintained in 5% CO_2_. Cell lines were purchased from ATCC (The Global Bioresource Center).

### Peptide binding

The binding and internalization of tracing-unit-conjugated NT4 was tested in MDA-MB 231 and SKOV-3 cell lines. 3 × 10^4^ cells/well were seeded on 24-well plates, grown for 24 hours, blocked for 30 min at 37 °C with 1% BSA in TBS and then incubated with NT4-biotin for 30 min at 37 °C (1 μM in TBS-0.1% BSA), followed by incubation for 30 minutes with 0.5 μg/ml SA-FITC. Cells were then fixed with TBS 4% formalin and nuclei were stained with DAPI (0.5 μg/ml in TBS-0.3% BSA). Each step was followed by three washes in TBS. Peptide binding was analysed by confocal laser microscope (Leica TCS SP5) with 380 λex and 450-470 λem, 488 λex and 530–550 λem for DAPI and FITC, respectively.

### PTX release

NT4-PTX and U4-PTX peptides (0.2 mM) were incubated at 37 °C with 3% Fetal Bovine Serum in growth medium. At different time intervals (T0, 1 hour and 3 hours) the mixtures were precipitated with methanol, diluted with 600 μl 0.1% trifluoroacetic acid (TFA)/water and analyzed by HPLC and mass spectrometry. Liquid chromatography was performed on a Phenomenex Jupiter C18 analytical column (300 Å, 5 μm, 250 × 4.6 mm), using 0.1% TFA/water as solvent A and methanol as solvent B with a linear gradient from 80% A to 5% A in 30 min. MS analysis of samples was performed with a Bruker Daltonic ultraflex MALDI TOF/TOF mass spectrometer.

### Cytotoxicity of Paclitaxel-conjugated branched NT

MDA-MB 231 and SKOV-3 cells were plated at a density of 5 × 10^3^ per well in 96-well microplates. Different concentrations of free or NT4-PTX, from 128 pM to 10 μM, were added 24 h after plating. Cells were washed after 1 h incubation and then left 6 days at 37 °C with the same medium. Growth inhibition was assessed by 3-(4,5-dimethylthiazol-2-yl)-2,5-diphenyltetrazolium bromide (MTT). Cytotoxicity of drug-conjugated NT4 was compared with that of the corresponding free drugs and of U4-PTX. The experiment was performed twice in triplicate.

EC50 values were calculated by non-linear regression analysis using Graph Pad Prism 5.03 software. Values from untreated controls gave 100% cell viability. The level of significance is p < 0.05 for two-sided testing.

### Tumor growth inhibition studies

Orthotopic breast tumors were established by injecting 1 × 10^6^ MDA-MB 231-*luc2* cells into the fourth mammary fat pad of athymic nude mice (Harlan Laboratories). Tumor growth was monitored every four days by measuring two perpendicular diameters with caliber. Tumor volume (V) was calculated by the following equation: V = (a^2^ × b)/2, where “a” is the width of the tumor (small diameter), and b the length (large diameter), both in millimeters^22^.

Treatments were started when the tumors reached at least 80 mm^3^ in volume. Tumor-bearing mice were randomized into three groups (five mice in NT4-PTX and PTX groups and four mice in control group) and injected intravenously every 4 days with 500 μg NT4-PTX (2.86 μmol/Kg), 48.91 μg PTX in cremophor EL®/ethanol solution (2.86 μmol/Kg) and saline.

Mice were housed in filter-top cages with a 12-hour dark-light cycle. All had unlimited access to food and water throughout the duration of the study.

All experiments were conducted in accordance with laws and regulations for experiments and procedures (Directive 2010/63/EU). All experimentation on live vertebrates described in this article was approved by the Italian Ministry of Health (document no. 140/2009-B) and by CEL AOUS (24/02/2009). The animals were observed carefully; pain and distress were documented before, during and after the experimental procedure. We ensured that animals did not show any signs of suffering or disease (such as weight loss, abdominal distension, impaired movement). They were euthanized by experienced persons as determined by the experimental protocol or on an ad-hoc basis in the event of unforeseen side effects. We ensured that the procedure did not cause more than momentary, minimal pain or distress.

### Animal imaging

Tumor growth was also monitored by imaging luciferase reporter expression in tumor cells. To track MDA-MB 231-*luc2* cells, bioluminescent optical imaging was performed using a Photon Imager system (Biospace Lab, Paris, France) including a cooled charge-coupled device (CCD) camera. Prior to imaging, mice were anaesthetized by intraperitoneal (i.p.) injection of 275 mg/kg Avertin. Bioluminescent images were acquired for 3-minute total exposure, in ventral position, 5 minutes after i.p. injection of D-luciferin (150 mg/Kg, XenoLight RediJect D-Luciferin, Caliper Life Sciences, Villepinte, France). Bioluminescence was quantified by automatic measurement. ROI optical images were analyzed with M3 Vision software (Biospace Lab, Paris, France).

## Additional Information

**How to cite this article**: Brunetti, J. *et al.* Tumor-selective peptide-carrier delivery of Paclitaxel increases *in vivo* activity of the drug. *Sci. Rep.* **5**, 17736; doi: 10.1038/srep17736 (2015).

## Supplementary Material

Supplementary Information
