# Supplementary material for: Tumor-selective peptide-carrier delivery of Paclitaxel increases in vivo activity of the drug
Source: Sci Rep. 2015 Dec 2;5:17736. doi: 10.1038/srep17736 (PMC4667195; doi:10.1038/srep17736)
Supplement: Supplementary Information [file srep17736-s1.pdf]

Tumor-selective peptide-carrier delivery of Paclitaxel increases in vivo activity of the drug

Jlenia Brunetti[a*], Serena Pillozzi[b], Chiara Falciani[c], Lorenzo Depau[a], Eleonora Tenori[d], Silvia Scali[a], Luisa Lozzi[a], Alessandro Pini[a], Annarosa Arcangeli[b], Stefano Menichetti[d], Luisa Bracci[a].

[a] *Department of Medical Biotechnologies, University of Siena, Siena 53100, Italy*

[b] *Department of Experimental and Clinical Medicine, University of Florence, Florence, 50139 Italy*

[c] *SetLance srl, Toscana Life Sciences, Siena, 53100 Italy*

[d] *Department of Chemistry, University of Florence, Florence, 50019 Italy*

* corresponding author

Supplementary material

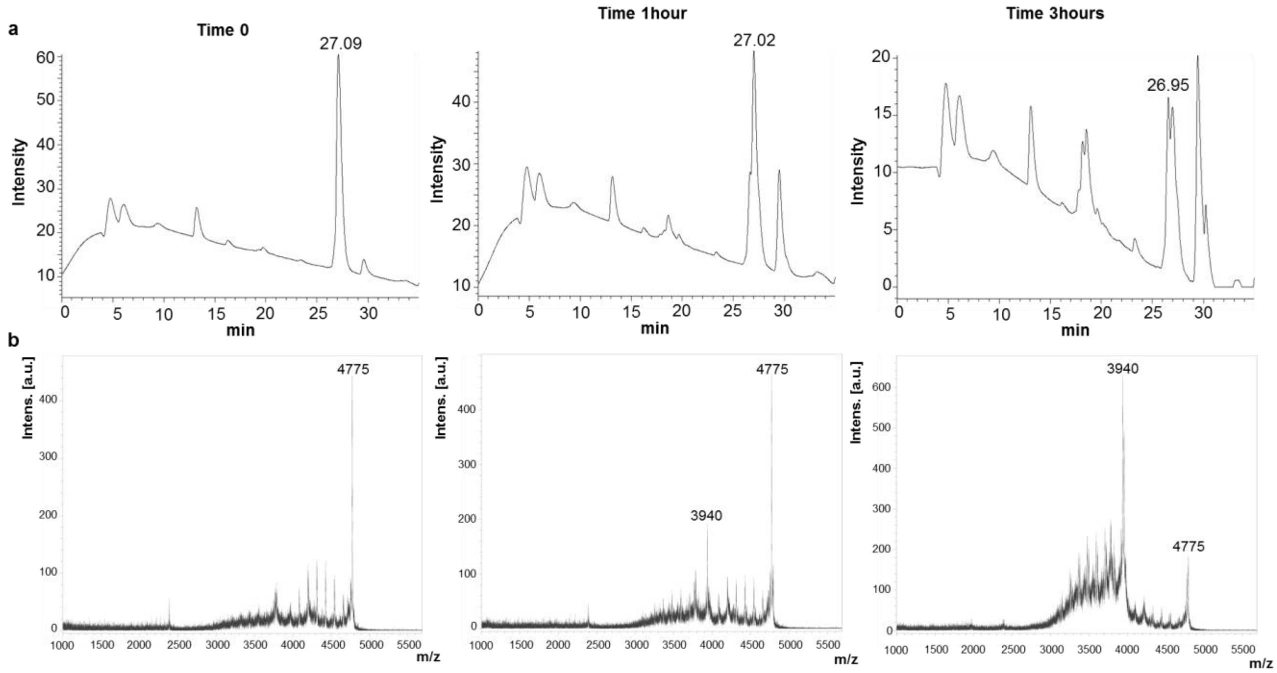

Figure S1. Paclitaxel release. HPLC **(a)** and MS **(b)** profiles of U4-PTX incubated with 3% serum at different times: 5 min, 1 hour and 3 hours.
